# Editorial: Rising stars in field robotics: 2022

**DOI:** 10.3389/frobt.2024.1379661

**Published:** 2024-02-14

**Authors:** Dimitrios Kanoulas, Shehryar Khattak, Giuseppe Loianno

**Affiliations:** ^1^ University College London, London, United Kingdom; ^2^ ETH Zürich, Zürich, Switzerland; ^3^ New York University, New York, NY, United States

**Keywords:** robotics, automation, legged robot, robot manipulation, LLM

## 1 Introduction

In an era where technological advancements shape our daily lives, recognizing emerging leaders in Robotics and Artificial Intelligence (AI) is not just important–it is imperative. “The Future Leaders of Robotics and AI” Research Topic serves this very purpose. Our aim is to showcase the exceptional work of internationally recognized researchers in the early stages of their careers, heralding a new age of innovation and discovery. This carefully curated Research Topic not only highlights groundbreaking research across the vast expanse of Field Robotics but also presents significant advances in theory, experiment, and methodology. The application of these studies addresses compelling problems, paving the way for future breakthroughs.

It is important to note that the selected papers are recognitions of talented early career researchers that might shape the trajectory of technological evolution. As we stand on the cusp of future innovations in the field of robotics, which remain largely undiscovered, this Research Topic offers a glimpse into who might lead these advancements. This initiative is more than just an academic exercise; it is a beacon for the scientific community and industry alike to identify and follow the luminaries of tomorrow.

In conclusion, while the full scope of future innovations in field robotics is yet to unfold, this Research Topic is a guiding light, pointing us towards the brilliant minds who will drive the next wave of technological revolution.

## 2 Overview of the contents of the Research Topic

Ghaffari et al., present advancements in the field of robot perception and control, focusing on the integration of symmetry in problem-solving. Drawing inspiration from mathematical techniques used to analyze symmetry in geometric spaces, this research explores how geometric sensor registration, state estimation, and control methods can offer crucial understanding in formulating robotics algorithms. These algorithms are particularly tailored for complex, unexplored environments. Furthermore, the incorporation of computational techniques for determining difficult-to-quantify factors enhances the efficacy of these symmetry-based methods. The paper substantiates its assertions by demonstrating experimental outcomes in real-world settings, covering aspects of robot perception, state estimation, and control.

Manoni et al., address the challenge of real-time adjustment in swarm flocking behaviors for aerial drones, a task complicated by fast, non-linear dynamics. Traditional methods using virtual potential functions for stability and velocity are inefficient in field conditions due to the need for extensive parameter tuning. They introduce a novel approach where each drone dynamically tunes local interactions, autonomously prioritizing various information sources like neighbor behavior or goal direction. A key innovation is the use of a Gaussian kernel to adjust the importance of each swarm element, aiding in precise control of output velocities. Their method not only achieves cohesive flocking but also navigates effectively through waypoints at high speeds. It further enables complex behaviors such as group splitting and joining over long distances. Extensive simulated and field experiments, in both communication-rich and communication-limited scenarios, demonstrate the robustness of their approach against failures, intermittent communication, and perception errors.

Muratore and Tsagarakis, explore the challenges in robotics for executing complex tasks in dynamic, unstructured environments like emergency response and search-and-rescue. These tasks require a delicate balance between computational power and energy autonomy. To address this, they introduce the XBot2D software architecture, which employs cloud robotics to enhance computational capabilities while conserving energy by offloading tasks to the cloud. However, real-world deployment is hindered by limitations in bandwidth, latency, and connectivity. XBot2D features a hybrid cloud manager that dynamically allocates robotic tasks between local and cloud resources, adjusting for network conditions and the robot’s computational and energy needs. This dual approach, encompassing both local and cloud computing, facilitates real-time operations on the robot and more complex AI and machine learning processes in the cloud, adapting automatically to performance requirements and network quality. The effectiveness of XBot2D is demonstrated through its application on the CENTAURO robot, integrated with Amazon Web Services EC2 cloud infrastructure under varying network conditions.

Chalvatzaki et al., explore the use of GPT-2, a smaller large language model (LLM), for long-horizon task planning in assistive and service robotics. They focus on the LLM’s ability to break down complex tasks into manageable subgoals for sequential execution by a robotic planner. By grounding the LLM’s input in a domain-specific scene graph, it effectively translates human instructions into actionable robot plans. This process aids the model in handling extensive tasks, as exemplified in the ALFRED benchmark. They evaluate their LLM-based planner against traditional planning methods and other baselines to assess its effectiveness and adaptability. The results indicate that the inherent knowledge in an LLM can be successfully applied to long-term task planning. This finding highlights the potential of integrating neuro-symbolic planning approaches in future robotic applications.

Cordie et al., address the challenge of actuator failure in remote robots, a problem that reduces efficiency and can lead to complete failure. As robots become more autonomous and operate beyond easy repair reach, resilience to such failures is crucial. They propose two strategies based on a modular robotic architecture to enhance robustness against actuator failures in both fixed and reconfigurable robots. Their approach involves modular reconfigurable robots that can alter their locomotion style and morphology by ejecting malfunctioning modules. This capability has shown to increase travel distance and reduce movement effort in both simulated and real-world environments. Robots with adaptive locomotion and morphology demonstrated significantly better resilience to actuator failure than those with fixed configurations. The findings are supported by tests conducted in gazebo simulations and field experiments, showing that the ability to discard failed components can notably extend mission duration. This research presents a pioneering approach to improving the operational lifespan of robots in challenging environments.

## 3 Conclusion

In conclusion, this Research Topic of research showcases significant advancements in the field of robotics and AI, addressing key challenges and introducing innovative solutions. Collectively, these studies not only push the boundaries of current robotic capabilities but also set the stage for future innovations in autonomous systems, proving indispensable for the progression of the field. Their contributions offer a glimpse into the future of robotics, where adaptability, efficiency, and intelligence converge to create more autonomous, reliable, and versatile robotic systems.

